# Double-burden of malnutrition among the indigenous peoples (*Orang Asli*) of Peninsular Malaysia

**DOI:** 10.1186/s12889-015-2058-x

**Published:** 2015-07-21

**Authors:** C. Y. Wong, M. S. Zalilah, E. Y. Chua, S. Norhasmah, Y. S. Chin, A. Siti Nur’Asyura

**Affiliations:** Department of Nutrition and Dietetics, Faculty of Medicine and Health Sciences, Universiti Putra Malaysia, 43400 UPM, Serdang, Selangor Darul Ehsan Malaysia

**Keywords:** Double-burden of malnutrition, Demographics and socio-economics, Indigenous peoples, *Orang Asli*

## Abstract

**Background:**

Double-burden of malnutrition (DBM) is an emerging public health concern among the *Orang Asli* (indigenous peoples) of Peninsular Malaysia. This study aimed to identify the presence of DBM at the community and household levels in *Orang Asli* population and its associated demographic and socio-economic factors.

**Methods:**

This cross-sectional study was conducted in 11 *Orang Asli* villages surrounding the Krau Wildlife Reserve, Peninsular of Malaysia from October 2011 to January 2012. Of 438 households, a total of 981 adults and 304 children who met the study criteria agreed to participate. About 160 households were further selected with pairs of children aged 3–59 months and non-pregnant mothers aged 15–55 years. Demographic and socio-economic data were obtained using interviewer-administered questionnaire while weight and height were measured using standard procedures. Double-burden of malnutrition was defined as overweight/obese mother-underweight child (OWOBM/UWC), overweight/obese mother-stunted child (OWOBM/STC) or overweight/obese mother-underweight or/and stunted child (OWOBM/UWSTC). Binary logistic regression identified the demographic and socio-economic factors associated with double-burden households.

**Results:**

About 26 % of overweight and obese adults coexisting with high proportions of underweight (49 %) and stunted (64 %) children in these *Orang Asli* villages. There was a higher prevalence of households with OWOBM/UWSTC (20 %) and OWOBM/STC (19.4 %) than households with OWOBM/UWC (12.5 %). Boys (*P* < 0.05), older age mothers (*P* < 0.05), mothers with higher education (*P* < 0.05) and households with income per capita less than USD 29.01 (RM 97.00) (*P* < 0.01) were associated with higher odds of OWOBM/STC. *Jah Hut* (*P* < 0.05) and higher number of children (*P* < 0.05) were significantly associated with lower odds of OWOBM/UWC.

**Conclusions:**

The occurrence of double-burden of malnutrition in *Orang Asli* population deserves attention. Poverty reduction, access to quality diet and improved health and nutrition literacy are among strategies that could address the coexistence of DBM in this population.

## Background

In many low- and middle-income countries, the persistence of undernutrition in children is accompanied by increasing overweight and obesity in older children, adolescents and adults. This phenomenon, known as “double-burden of malnutrition” (DBM), is emerging within countries, communities and even households [1–5]. National surveys from six low- to middle-income countries showed that 3.7–15.5 % of households were with underweight and overweight members [1], while national surveys from 42 developing countries in Africa, Asia and Latin America reported that there were 0.9–16.0 % of households with a stunted child and an overweight or obese mother [2]. This DBM phenomenon is an emergent public health problem that could lead to impaired child growth and cognitive development which in turn will dramatically increase the risk of obesity and non-communicable chronic diseases as well as impaired social and economic productivity in adulthood [6–8]. In addition, DBM profoundly complicates nutritional interventions because under- and over-nutrition have so far been treated as distinct nutritional problems when in fact they may share similar determinants [3, 9].

The DBM phenomenon is due to interaction of changes related to demographic and socio-economic status, traditional food patterns and lifestyle [10]. Studies have shown that DBM is more prevalent in urban than rural areas of developing countries, particularly among low income households, but gradually increasing in the rural areas [1, 3, 9, 11]. Factors such as household size, number of children, maternal (age, marital status, education and employment status) and child factors (age and sex) were also associated with risk of DBM households [3, 9, 11–13].

Indigenous peoples make up approximately 4.5 % (~300 million) of the global population and they are still among the poorest and most marginalized groups with poor health and social outcomes [14]. Indigenous peoples are not spared from DBM. For example, over half of the indigenous adults in Australia were overweight and obese despite the persistence of undernutrition in children (10–14 %) [15]. Likewise, the First National Survey of Indigenous Peoples in Brazil reported that about 6 % and 26 % of children were underweight and stunted, while 46.1 % of adults were overweight and obese [16, 17]. The increasing prevalence of DBM phenomenon is parallel to the rising prevalence of diabetes mellitus, hypertension and cardiovascular diseases among indigenous peoples in Brazil [17], Australia [18, 19] and Canada [20].

The indigenous peoples of Peninsular Malaysia (*Orang Asli*) are also experiencing a high rate of obesity, hypertension and diabetes even though they contribute to 0.7 % of the total population in Peninsular Malaysia [21]. Several studies showed that approximately 10–35 % of *Orang Asli* adults in Peninsular Malaysia were overweight and obese, yet more than half of the children were underweight and stunted [22–25]. In an earlier study, the prevalence of diabetes mellitus (1.3 %) and impaired glucose tolerance (10.7 %) was significantly higher among *Orang Asli* living in rural area than those living in the remote forest (0.0 % and 3.3 % respectively) [26]. Wan Nazaimoon & Suraiami [27] reported that 22.7 % of female *Orang Asli* adults in Peninsular Malaysia had metabolic syndrome, with the prevalence of diabetes mellitus and impaired fasting glucose was 8.4 % and 16.8 %, respectively. A recent study among the *Orang Asli* of Peninsular Malaysia showed that the prevalence of obesity, abdominal obesity, hypertension and diabetes mellitus was 16.8 %, 38.4 %, 29.6 % and 4.6 %, respectively [28].

While undernutrition still prevails in *Orang Asli* children [22, 25], obesity and metabolic risks are increasingly prevalent among the *Orang Asli* adults. This study aimed to assess the presence of double-burden of malnutrition at the community and household levels by assessing the nutritional status of *Orang Asli* adults and children. In addition, we further investigated the demographic and socio-economic characteristics that differentiate normal and double-burden of malnutrition households in this community.

## Methods

### Study design and location

This cross-sectional study was conducted in Krau Wildlife Reserve (KWR) from October 2011 to January 2012. The reserve covering 62,395 hectares is located near the Benom Mountain in central state of Pahang, Malaysia [29]. According to the Malaysia Department of Orang Asli Development [30], there are 11 *Orang Asli* villages around the KWR with approximately 3,636 individuals living in 833 households. The major *Orang Asli* sub-tribes living in these villages are *Jah Hut* and *Che Wong* of *Senoi* ethnic group, and *Temuan* of *Proto Malay* ethic group [30].

### Study subjects

A total of 533 households were identified from the 11 *Orang Asli* villages around KWR but only 485 households agreed to participate in this study. The other 300 households could not be visited due to lack of accessibility to the households (i.e. residing in the forest interior or at the other side of Kuala Krau River) or households no longer exist.

There were two phases of study. In the first phase (community level), a total of 1,521 adults aged 18 years and above and 363 children below 5 years of *Jah Hut*, *Temuan* and *Che Wong* tribes, with no health disabilities from the 485 households were identified. Pregnant women and mothers who gave birth less than 3 months prior to the measurements were excluded. However, only 981 adults and 304 children completed weight and height measurements. In the second phase (household level), the 485 households were further screened for mother-child pairs. A total of 160 mother-child pairs met the inclusion criteria of at least one child (6–59 months of age) and non-pregnant mother (15–55 years of age). For households with more than one mother-child pairs, only one pair was randomly selected. In addition, if a family has 2 or more children in the age range of 6–59 months, the oldest one was selected to the mother-child pair (index child).

### Measurements

#### Demographic and socio-economic characteristics

Information on household characteristics (e.g. ethnicity, household size, number of children, number of schooling children and household income), maternal (e.g. age, marital status, education, occupation, income) and child profile (e.g. sex, age) were obtained from household heads (mainly fathers) or mothers, using a set of pre-tested administered questionnaire. Information on age was recorded based on identification cards or birth certificates.

#### Physical measurement

Weight and height measurements were taken using standard procedures [31]. Tanita digital weighing scale (Tanita Corporation, Tokyo, Japan) and SECA stadiometer (Vogel and Halke & Co., Hamburg, Germany) were used to measure weight and height of children (2 years and above) and adults, to the nearest 0.1 kg and 0.1 cm, respectively. A SECA model 334 infantometer (SECA Gmgh & Co. Kg, Hamburg, Germany) was used to measure the recumbent weight and length of infants and young children (less than 2 years), to the nearest 0.01 kg and 0.1 cm, respectively. Age- and sex-specific z-scores for weight-for-age (WAZ), height-for-age (HAZ) and body mass index-for-age (BAZ) of children (below 5 years) were calculated using WHO Anthro software (version 3, 2009). Underweight, stunting and thinness for children were defined as WAZ, HAZ and BAZ < - 2 standard deviation (SD) whereas overweight for children were defined as BAZ ≥ 2 standard deviation [32]. Body mass index (BMI) of adults was calculated from the weight and height measurements. Underweight and overweight/obesity for mother were defined as BMI < 18.5 kg/m^2^ and BMI > 24.99 kg/m^2^, respectively [31].

#### Classifications of the *Orang Asli*’s nutritional status at community and household levels

Weight-for-age (WAZ) and height-for-age (HAZ) for children and body mass index (BMI) for adults and mothers were used to assess nutritional status of the *Orang Asli* at the community and household levels. At the community level, we defined DBM as having a high prevalence of stunting/underweight coexisting with a high prevalence of overweight/obesity within a community/population [1, 33]. The “high prevalence” was defined as any value greater than the expected prevalence of 15 % for overweight/obesity and 5 % for stunting based upon the range of BMI and height in the reference or standard populations [34]. At the household level, we defined DBM as the coexistence of an overweight or obese mother (OWOBM) and an underweight and/or stunted child (UWSTC) within the same household. By using the 2 indicators of WAZ and HAZ for children and BMI for mothers, the 6 classifications of nutritional status of mother-child pairs are presented in Table 1.

**Table 1:** Classifications of nutritional status of mother-child pairs

Classification	BMI_mother_ vs. WAZ_child_	BMI_mother_ vs. HAZ_child_	BMI_mother_ vs. WAZ/HAZ_child_
1. Double burden household	OWOBM/UWC	OWOBM/STC	OWOBM/UWSTC
2. Mother normal/child normal	NWM/NWC	NWM/NHC	NWM/NWNHC
3. Mother normal/child undernutrition	NWM/UWC	NWM/STC	NWM/UWSTC
4. Mother overweight or obesity/child normal	OWOBM/NWC	OWOBM/NHC	OWOBM/NWNHC
5. Mother underweight/child normal	UWM/NWC	UWM/NHC	UWM/NWNHC
6. Mother underweight/child undernutrition	UWM/UWC	UWM/STC	UWM/UWSTC

*OWOBM* overweight or obese mother, *NWM* normal weight mother, *UWM* underweight mother, *UWC*, underweight child, *NWC* normal weight child, *STC* stunted child, *NHC* normal height child, *UWSTC* underweight or/and stunted child, *NWNHC* normal weight and height child

### Ethics approval

The study protocol was approved by the Medical Research Ethics Committee of the Faculty of Medicine and Health Sciences, Universiti Putra Malaysia. Permission to conduct this study was obtained from the Malaysia Department of Orang Asli Development (JAKOA) and Department of Wildlife and National Parks Peninsular Malaysia (PERHILITAN). Prior to data collection, researchers verbally informed household heads about the study and obtained initialized or verbal informed consent from household heads or mothers.

### Statistical analysis

IBM SPSS Statistics 20.0 (IBM Corporation, New York, USA) was used for data analysis. All variables were presented as mean, standard deviation and frequency. Preliminary analysis included the descriptive statistics on nutritional status of adults and children, which was used to assess the presence of DBM at the community and household levels. Binary logistic regression models were further used to identify the demographic and socio-economic factors associated with DBM households (OWOBM/UWC: overweight or obese mother/underweight child; OWOBM/STC: overweight or obese mother/stunted child; OWOBM/UWSTC: overweight or obese mother/underweight or/and stunted child). The reference group for all analyses was normal households (NWM/NWC: normal weight mother and child; NWM/NHC: normal weight mother/normal height child; NWM/NWNHC: normal weight mother/normal weight and height child). In the binary logistic regression models, univariate logistic regression analysis was first used to assess the association of independent variable individually with the dichotomous dependent variable. Results were presented as odds ratios (OR) with 95 % confidence interval (CI). Then, we further estimated the adjusted odds ratios (adj. OR) by adding the independent variables that were significant at univariate level (lenient cut-off point of *P* < 0.25) or that needed to be included in order to control for confounding, in multivariate analysis. Model fit was determined by the Hosmer-Lemeshow statistic. Statistical significance was set at *P* < 0.05.

## Results

### Demographic and socio-economic characteristics

A total of 981 adults (86.7 % *Jah Hut*, 7.0 % *Temuan* and 6.2 % *Che Wong*) from 485 *Orang Asli* households participated in this study (Table 2). This study comprised of 46 % men and 54 % women with a mean age of 37.3 ± 14.9 years. The mean year of schooling was 3.7 ± 4.2 years. More than 70 % of households had per capita monthly income below the poverty line for Peninsular Malaysia of USD 58.02 (RM 194) [35]. The 160 *Orang Asli* households with mother-child pairs comprised of 87.5 % *Jah Hut*, 4.4 % *Temuan* and 8.1 % *Che Wong* (Table 2). There were 59% and 41 % of boys and girls, respectively with a mean age of 33.7 ± 16.4 months. The mean age of mothers was 29.7 ± 7.4 years with a majority (88 %) in the age range of 18–39 years. Most mothers had low educational level (4.1 ± 3.6 years) and were housewives or unpaid family workers (91 %).

**Table 2:** Demographic and socio-economic characteristics of *Orang Asli* at community and household levels

Characteristic	*n* (%)	*Mean ± SD*
*Community level: adults (N = 981)*		
Ethnicity		
*Jah Hut*	851 (86.7)	
*Temuan*	69 (7.0)	
*Che Wong*	61 (6.2)	
Sex		
Male	455 (46.4)	
Female	526 (53.6)	
Age (years)		37.3 ± 14.9
Marital status		
Single	197 (20.1)	
Married	678 (69.1)	
Divorced/widow	106 (10.8)	
Education (years)		3.7 ± 4.2
Employment		
Employed (employer, employee, own account worker)	459 (46.8)	
Unpaid family worker/housewife	374 (38.2)	
Unemployed/student	99 (10.1)	
Retired	49 (5.0)	
Income (RM/month)		432.65 ± 361.12
Household size		6.9 ± 3.2
Number of children		3.0 ± 2.2
Number of schooling children		1.5 ± 1.6
Household income (RM/month)		853.32 ± 880.23
Household income per capita (RM/month)^a^		130.61 ± 120.04
< USD 29.01 (< RM 97)	497 (50.7)	
USD 29.01–58.02 (RM 97–194)	273 (27.8)	
≥ USD 58.02 (≥ RM 194)	211 (21.5)	
*Household level*: *mother*-*child pairs* (*N* = *160*)		
Ethnicity		
*Jah Hut*	140 (87.5)	
*Temuan*	7 (4.4)	
*Che Wong*	13 (8.1)	
Child’s sex		
Boys	94 (58.8)	
Girls	66 (41.3)	
Child’s age (months)		33.7 ± 16.4
Maternal age (years)		29.7 ± 7.4
Maternal marital status		
Married	152 (95.0)	
Single/divorced/widow	8 (5.0)	
Maternal education (years)		4.0 ± 3.6
Maternal employment		
Housewife/unpaid family worker	145 (90.7)	
Employed	15 (9.4)	
Maternal income (RM/month)		264.06 ± 234.34
Household size		7.3 ± 3.3
Number of children		3.7 ± 2.1
Number of schooling children		1.7 ± 1.8
Household income (RM/month)		851.37 ± 779.86
Household income per capita (RM/month)^a^		120.24 ± 93.38
< USD 29.01 (< RM 97)	83 (51.9)	
USD 29.01–58.02 (RM 97–194)	47 (29.4)	
≥ USD 58.02 (≥ RM 194)	30 (18.8)	

^a^Less than USD 58.02 (RM 194) (capita/month) is classified as household living in poverty according to the government Poverty Income Line [35]

USD 1 = RM 3.30

### Nutritional status: the occurrence of double-burden of malnutrition

At the community level, about 13 %, 19 % and 7 % of the adults were underweight, overweight and/or obese, respectively (Table 3). While 17 % of mothers were underweight, there were more than one-fourth of mothers (28 %) were overweight and obese. In addition, high proportions of children were stunted (64.2 %) and underweight (48.7 %). Stunting (67.3 % vs. 60.5 %) and underweight (54.4 % vs. 42.3 %) were more prevalent in boys than girls. More children were reported as thin (11.9 %) than at risk of overweight (3.9 %) and overweight (1.3 %).

**Table 3:** Nutritional status of *Orang Asli* at community and household levels

Characteristic	Male	Female	Total
*Community level: nutritional status*	*n* = 455	*n* = 526	*N* = 981
**Adults**			
Weight (kg) (*Mean ± SD*)	57.9 ± 11.7	50.0 ± 12.7	53.7 ± 12.9
Height (cm) (*Mean ± SD*)	159.1 ± 6.5	147.7 ± 6.2	153.0 ± 8.5
Body mass index (BMI, kg/m^2^) (*Mean ± SD*)	22.8 ± 4.1	22.8 ± 4.9	22.8 ± 4.6
Underweight (< 18.50)	38 (8.4)	89 (16.9)	127 (12.9)
Normal (18.50–24.99)	310 (68.1)	294 (55.9)	604 (61.6)
Overweight (25.00–29.99)	80 (17.6)	101 (19.2)	181 (18.5)
Obese (≥ 30.00)	27 (5.9)	42 (8.0)	69 (7.0)
**Mothers of children below 5 years**			*N* = 205
Weight (kg) (*Mean ± SD*)			52.0 ± 11.0
Height (cm) (*Mean ± SD*)			148.7 ± 4.9
Body mass index (BMI, kg/m^2^)			23.5 ± 4.3
Underweight (< 18.50)			14 (6.8)
Normal (18.50–24.99)			128 (62.4)
Overweight (25.00–29.99)			49 (23.9)
Obese (≥ 30.00)			14 (6.9)
**Children below 5 years**	*n* = 162	*n* = 142	*N* = 304
Weight-for-age (WAZ) (*Mean ± SD*)	−2.0 ± 1.2	−1.7 ± 1.1	−1.9 ± 1.2
Severe underweight(< −3.0 SD)	32 (19.8)	24 (16.9)	56 (18.4)
Moderate underweight (−3.0 to < −2.0 SD)	56 (34.6)	36 (25.4)	92 (30.3)
Normal (−2.0 to 2.0 SD)	74 (45.7)	82 (57.7)	156 (51.3)
Height-for-age (HAZ) (*Mean ± SD*)	−2.5 ± 1.3	−2.3 ± 1.2	−2.4 ± 1.2
Severe stunting (< −3.0 SD)	55 (34.0)	34 (23.9)	89 (29.3)
Moderate stunting (−3.0 to < −2.0 SD)	54 (33.3)	52 (36.6)	106 (34.9)
Normal (−2.0 to 2.0 SD)	53 (32.7)	56 (39.4)	109 (35.9)
Body mass index-for-age (BAZ) *(Mean ± SD*)	−0.6 ± 1.1	−0.6 ± 1.1	−0.6 ± 1.1
Severe thinness (< −3.0 SD)	4 (2.5)	2 (1.4)	6 (2.0)
Moderate thinness (−3.0 to < −2.0 SD)	18 (11.1)	12 (8.5)	30 (9.9)
Normal (−2.0 to < 1.0 SD)	133 (82.1)	119 (83.8)	252 (82.9)
At risk overweight (1.0 to < 2.0 SD)	5 (3.1)	7 (4.9)	12 (3.9)
Overweight (2.0 and above)	2 (1.2)	2 (1.4)	4 (1.3)
*Household level: classification of nutritional status of mother-child pairs*			*N* = 160
BMI_mother_ vs. WAZ_child_			
NWM/NWC			41 (25.6)
OWOBM/UWC			20 (12.5)
OWOBM/NWC			34 (21.3)
UWM/UWC			7 (4.4)
UWM/NWC			2 (1.3)
NWM/UWC			56 (35.0)
BMI_mother_ vs. HAZ_child_			
NWM/NHC			20 (12.5)
OWOBM/STC			31 (19.4)
OWOBM/NHC			23 (14.4)
UWM/STC			7 (4.4)
UWM/NHC			2 (1.3)
NWM/STC			77 (48.1)
BMI_mother_ vs. WAZ/HAZ_child_			
NWM/NWNHC			17 (10.6))
OWOBM/UWSTC			32 (20.0)
OWOBM/NWNHC			22 (13.8)
UWM/UWSTC			8 (5.0)
UWM/NWNHC			1 (0.6)
NWM/UWSTC			80 (50.0)

*NWM/NWC* normal weight mother/normal weight child, *OWOBM/UWC* overweight or obese mother/underweight child, *OWOBM/NWC* overweight or obese mother/normal weight child, *UWM/UWC* underweight mother/underweight child, *UWM/NWC* underweight mother/normal weight child, *NWM/UWC* normal weight mother/underweight child, *NWM/NHC* normal weight mother/normal height child, *OWOBM/STC* overweight or obese mother/stunted child, *OWOBM/NHC* overweight or obese mother/normal height child, *UWM/STC* underweight mother/stunted child, *UWM/NHC* underweight mother/normal height child, *NWM/STC* normal weight mother/stunted child, *NWM/NWNHC* normal weight mother/normal weight and height child, *OWOBM/UWSTC* overweight or obese mother/underweight or/and stunted child, *OWOBM/NWNHC* overweight or obese mother/normal weight and height child, *UWM/UWSTC* underweight mother/underweight or/and stunted child, *UWM/NWNHC* underweight mother/normal weight and height child, *NWM/UWSTC* normal weight mother/underweight or/and stunted child

Households with a normal mother paired with an underweight/stunted child (NWM/UWSTC) (50 %) were found to be most prevalent in the *Orang Asli* communities (Table 3). The coexistence of overweight/obese mother and underweight/stunted child (OWOBM/UWSTC) was observed in 20 % of the *Orang Asli* households. There was a higher prevalence of households with overweight/obese mother-stunted child (OWOBM/STC) (19.4 %) than households with overweight/obese mother-underweight child (OWOBM/UWC) (12.5 %).

### Demographic and socio-economic factors associated with double-burden of malnutrition among mother-child pairs

Factors that are significantly associated with DBM households, defined by mother overweight or obese/child underweight (OWOBM/UWC) include ethnicity and number of children (Table 4). *Jah Hut* (adj. OR: 0.1, *P* < 0.05) and higher number of children (adj. OR: 0.3, *P* < 0.05) were significantly associated with lower odds of DBM. For DBM households, defined as mother overweight or obese/child stunted (OWOBM/STC), boys (adj. OR: 8.5, *P* < 0.05), older age mothers (adj. OR: 1.2, *P* < 0.05), mothers with higher education (adj. OR: 1.7, *P* < 0.05) and households with income per capita less than USD 29.01 (RM 97.00) (adj. OR: 16.8, *P* < 0.01) were associated with higher odds of DBM (Table 4). Boys (adj. OR: 7.2, *P* < 0.05), mothers with higher education (adj. OR: 1.5, *P* < 0.05) and households with income per capita less than USD 29.01 (RM 97.00) (adj. OR: 11.1, *P* < 0.05) were significantly associated with DBM households, defined as mother overweight or obese/child underweight or stunted (OWOBM/UWSTC) (Table 4).

**Table 4:** Association of demographic and socio-economic characteristics with 3 different definitions of mother-child with double-burden of malnutrition

Demographic and socio-economic characteristics	OWOBM/UWC (*n* = 20)^a^		OWOBM/STC (*n* = 31)^b^		OWOBM/UWSTC (*n* = 32)^c^	
	Crude OR (95 % CI)	Adjusted OR (95 % CI)	Crude OR(95 % CI)	Adjusted OR (95 % CI)	Crude OR (95 % CI)	Adjusted OR (95 % CI)
Ethnicity^e^						
*Jah Hut*	0.2 (0.0–1.2)	0.1 (0.0–0.6)*	0.8 (0.1–4.5)	0.7 (0.1–5.7)	0.4 (0.1–4.3)	0.4 (0.0–5.1)
*Temuan* and *Che Wong* ^d^	1.0	1.0	1.0	1.0	1.0	1.0
Child’s sex^f^						
Boys	1.5 (0.5–4.4)	2.4 (0.6–10.3)	1.9 (0.6–6.1)	8.5 (1.3–53.0)*	1.9 (0.6–6.2)	7.2 (1.2–44.4)*
Girls^d^	1.0	1.0	1.0	1.0	1.0	1.0
Child’s age (months)^f^	1.0 (1.0–1.1)*	1.1 (1.0–1.1)	1.0 (1.0–1.1)	1.0 (0.9–1.0)	1.0 (1.0–1.1)	1.0 (1.0–1.0)
Maternal age (years)^g^	1.1 (1.0–1.1)	1.0 (0.9–1.2)	1.1 (1.0–1.2)	1.2 (1.0–1.4)*	1.1 (1.0–1.1)	1.2 (1.0–1.3)
Maternal education^g^	0.9 (0.8–1.1)	1.0 (0.8–1.3)	1.0 (0.8–1.2)	1.7 (1.1–2.6)*	1.0 (0.8–1.2)	1.5 (1.0–2.3)*
No of children^h^	1.1 (0.8–1.4)	0.3 (0.1–0.9)*	1.3 (1.0–1.8)	1.1 (0.5–2.6)	1.2 (0.9–1.6)	1.0 (0.4–2.2)
Household income per capita^g^						
< USD 29.60 (< RM 97.00)	1.3 (0.4–3.8)	1.9 (0.5–7.9)	4.2 (1.3–14.2)*	16.8 (2.4–117.3)**	3.1 (0.9–10.4)	11.1 (1.7–75.1)*
≥ USD 29.60 (≥ RM 97.00)^d^	1.0	1.0	1.0	1.0	1.0	1.0

*OWOBM/UWC* overweight or obese mother/underweight child, *NWM/NWC* normal weight mother/normal weight child, *OWOBM/STC* overweight or obese mother/stunted child, *NWM/NHC* normal weight mother/normal height child, *OWOBM/UWSTC* overweight or obese mother/underweight or/and stunted child, *NWM/NWNHC* normal weight mother/normal weight and height child

USD 1 = RM 3.30

*P < 0.05; **P < 0.01

Reference group for outcome/dependent variables:

^a^NWM/NWC (*n* = 41)

^b^NWM/NHC (*n* =20)

^c^NWM/NWNHC (*n* = 17)

^d^Reference group for independent variables

^e^Ethnicity variable was adjusted for household size, no of children, no of schooling children and household income per capita

^f^Each variable was adjusted for child’s sex and age, and maternal age and education

^g^Each variable was adjusted for child’s sex and age, maternal age and education, household size, no of children and household income per capita

^h^No of children variable was adjusted for child’s sex and age, ethnicity, household size, no of schooling children and household income per capita

## Discussion

Similar to other indigenous communities worldwide [15, 17, 25], DBM occurs in the *Orang Asli* community of the current study, with a high prevalence of adult overweight/obesity coexisting with a very high prevalence of child undernutrition. This DBM phenomenon could be partially due to high proportions of stunted children and short stature of adults (i.e. women) that were observed in this *Orang Asli* community. In Guatemala, more indigenous women were reported to have short stature compared to non-indigenous women [36] and maternal short stature was significantly associated with overweight mother-stunted child pairs [9, 11]. The intergenerational transmission of stunting from mother to child, in which poor intrauterine growth brought on by a malnourished/stunted mother is associated with low birth weight and stunting in early childhood [6–8]. Another possible explanation for DBM is that indigenous populations are undergoing nutrition transition [37], whereby they experience a shift from highly mobile lifestyle (subsistence farming and hunting-fishing-gathering) and traditional diets high in plant-source foods to westernized diets high in fat, sugars, salt and animal-source foods, and sedentary lifestyle [15, 25, 28, 38, 39]. These changes could contribute to child undernutrition and adult overweight/obesity due to impaired control of dietary energy, lower energy expenditure and a lower rate of fat oxidation [40].

At household level, we reported a higher prevalence of households with OWOBM/UWSTC (20 %) and OWOBM/STC (19 %) than OWOBM/UWC (13 %). Similarly, Lee et al. [11] reported that 21.5 % of indigenous households in Guatemala comprised of OWOBM/STC. Several other studies have reported lower prevalence of double-burden households. In poor urban area of Benin (West Africa), the prevalence of households with maternal overweight/obesity and child stunting/wasting was 16.2 % [41] while households with OWOBM/STC in rural Indonesia and Bangladesh were 11 % and 4 %, respectively [9]. Jehn & Brewis [3] showed that there were less than 10 % of households with OWOBM/STC in the middle and lower income countries, except in Egypt (12.5 %), Ghana (12.5 %), Nicaragua (12.5 %), Bolivia (15 %), Peru (16 %) and Guatemala (23 %). For households with OWOBM/UWC, the reported prevalence was lower than the prevalence reported in previous local studies [12, 13, 42]. Saibul et al. [12] reported that 25.8 % of *Orang Asli* households in semi-urban areas had OWOBM/UWC while 26–29.6 % of Malay households in rural and semi-urban areas were with OWOBM/UWC [13, 42]. While a study among urban poor in Haiti (14.3 %) [43] reported a similar prevalence, the Demographic and Health Surveys (DHS) database from 18 middle and lower income countries in South Asia, Africa and Latin America (0.3–5.3 %) [3] showed much lower prevalence of households with OWOBM/UWC as compared to the present study. The different prevalence of DBM across studies could be due to the different proportions of maternal overweight and child underweight/stunting across regions, countries or populations.

While several studies showed that DBM was associated with higher income and wealthier households [1, 9], others have reported that DBM was prevalent among the lower and middle income households [11, 42]. Similarly, we observed that double-burden households with OWOBM/STC and OWOBM/UWSTC were more likely to occur in lower income households (households income per capita < USD 29.01). Inadequate resources to purchase quality foods may contribute to the purchase of cheaper energy-dense and nutrient-poor foods by lower income households to maintain families’ daily dietary intakes at less cost [44]. The consumption of these foods may predispose children to poor growth and development due to inadequate important nutrients and energy, particularly if small amounts are consumed, but may provide excess calories for adults [37].

We found that the OWOBM/UWC households were more prevalent in the non-*Jah Hut* (*Temuan* and *Che Wong*). For *Jah Hut*, while there is increasing dependence on rubber/oil palm cultivation, subsistence farming and hunting-fishing-gathering are still important food activities [25]. The involvement of *Jah Hut* women in these activities may sustain the dependence on traditional food sources and physical activity level. Living near to the town area might explain the easier access to processed foods by the *Che Wong* and *Temuan*, although the *Che Wong* (mostly men) still maintains hunting-fishing-gathering activities [24, 38]. Studies have shown that *Che Wong* and *Temuan* tend to depend on relatively inexpensive processed foods (e.g. sweetened condensed milk, sugar and cooking oil) that can be purchased at the nearest sundry shops/town when income is inadequate and forest resources for food and economy dwindle due to the monsoon season [12, 24, 39].

In the present study, boys were more likely to be in DBM households with OWOBM/STC and OWOBM/UWSTC. However, several studies demonstrated that female children were more likely to be in households with OWOBM/STC [4, 9]. A plausible explanation for the inconsistent findings is sex differences in child nutritional status across countries. For example, boys were more likely to be stunted than girls in Sub-Saharan Africa [45] whereas girls tend be more stunted than boys in Bangladesh [46]. A higher stunting prevalence among boys than girls in the present sample might explain the association between DBM and male child gender. In addition, cultural influence on child feeding and caring due to power relations and social norms that perpetuate discriminatory attitudes and practices may also contribute to the sex differences in DBM [47].

Several maternal characteristics were associated with DBM, including higher maternal education and older mother. Numerous studies have reported that higher maternal education was protective against DBM [3, 9, 11, 42, 48]. However, the present study and a study in rural Bangladesh [9] showed that double-burden of OWOBM/STC was more likely to occur in households where mothers had higher education level. The conflicting findings underscore the complexities of the association between maternal education and DBM. Maternal education may not necessarily be sufficient to initiate behavioral change related to health and nutrition [49]. Lacking of health and nutrition knowledge could contribute to women being less sensitive to child and own health or less responsive to health and nutrition issues due to barriers such as food cost, accessibility and availability, lack cooking skill and equipment [44, 49]. On the other hand, our findings, together with previous studies [3, 9] showed that older mothers were associated with double-burden of OWOBM/STC. Reduced physical activity due to physical exertion decreases with age may contribute to overweight and obesity among older mothers in DBM households [10].

We showed that OWOBM/UWC was less likely to occur in households with higher number of children. In contrast, Jehn & Brewis [3] reported that having more children in the households was associated with increased risk of OWOBM/UWC. However, it should be noted that we considered children < 18 years while Jehn & Brewis [3] investigated young siblings < 5 years. Different age ranges in defining “number of children in the same household” in both studies may explain these different findings. We also found that in this sample, number of children in the household increased with household size, number of schooling children and household income (data not shown). As many of schooling *Orang Asli* children are staying at school hostel during school terms, the number of children living in the household is lesser which could mean more resources for family members (i.e., food, time and money) [48]. It could also mean that having many children, particularly older ones, provides extra hands to care for younger siblings or contribute to household income.

There are several limitations to the present study. The cross-sectional study design to investigate the association of demographic and socio-economic factors with DBM could not determine the cause and effect relationship. In addition the small sample size, inclusion of only three sub-tribes and specific study location could limit the generalization of study findings to indigenous peoples of Peninsular Malaysia. The small sample size could also limit additional multivariate analyses to compare DBM households with other household phenotypes and limit the number of variables that could be included in the multivariate models. The present study did not examine other important determinants of DBM such as dietary intake, physical activity, caregiving practices, cultural influences, pregnancy and birth information. Finally, as the operational definition of “double-burden of malnutrition” varied across studies, comparing the prevalence and determinants of DBM with other countries and within different contexts is inevitably difficult. Despite these limitations, the present study has provided important information on DBM in indigenous communities and its socio-demographic characteristics which could be the basis for strategies to improve health and nutrition of the *Orang Asli*.

## Conclusions

DBM is a public health concern in lower income communities undergoing nutrition and socio-economic transition, and the *Orang Asli* population in Peninsular Malaysia is not spared. Undernutrition in early childhood needs to be addressed as underweight or/and stunted children may be predisposed to be overweight and obese in adulthood [50, 51]. The insights on demographic and socio-economic factors associated with DBM underscore the importance of having effective and culturally sensitive strategies to simultaneously address undernutrition and over-nutrition in children and adults, respectively. Poverty reduction, access to quality diet and improved health and nutrition literacy would be important and useful strategies to address the coexistence of DBM in this population.

## Abbreviations

DBM: Double-burden of malnutrition
KWR: Krau Wildlife Reserve
PERHILITAN: Department of Wildlife and National Parks Peninsular Malaysia
JAKOA: Department of Orang Asli Development
WAZ: Weight-for-age
HAZ: Height-for-age
BAZ: Body mass index-for-age
SD: Standard deviation
BMI: Body mass index
OWOBM: Overweight or obese mother
NWM: Normal weight mother
UWM: Underweight mother
STC: Stunted child
NHC: Normal height child
UWC: Underweight child
NWC: Normal weight child
STUWC: Stunted or/and underweight child
NHNWC: Normal height and weight child
OWOBM/STC: Overweight or obese mother/stunted child
OWOBM/UWC: Overweight or obese mother/underweight child
OWOBM/UWSTC: Overweight or obese mother/underweight and/or stunted child
NWM/NHC: Normal weight mother/normal height child
NWM/NWC: Normal weight mother and child
NWM/NWNHC: Normal weight mother/normal weight and height child
OR: Odds ratios
Adj. OR: Adjusted odds ratios
CI: Confidence interval
RM: Ringgit Malaysia
USD: United States Dollar
WHO: World Health Organization
